# Dynamic Circulation and Genetic Exchange of a Shrew-borne Hantavirus, Imjin virus, in the Republic of Korea

**DOI:** 10.1038/srep44369

**Published:** 2017-03-15

**Authors:** Seung-Ho Lee, Won-Keun Kim, Jin Sun No, Jeong-Ah Kim, Jin Il Kim, Se Hun Gu, Heung-Chul Kim, Terry A. Klein, Man-Seong Park, Jin-Won Song

**Affiliations:** 1 Department of Microbiology, College of Medicine, Korea University, Seoul, 02841 Republic of Korea; 2 5th R&D Institute, Agency for Defense Development, Daejeon, 34186 Republic of Korea; 3 5th Medical Detachment, 168th Multifunctional Medical Battalion, 65th Medical Brigade, Unit 15247, APO AP 96205-5247, United States of America; 4 MEDDAC-Korea, 65th Medical Brigade, Unit 15281, APO AP 96205-5281, United States of America

## Abstract

Hantaviruses (family *Bunyaviridae*) are enveloped negative-sense tripartite RNA viruses. The natural hosts of hantaviruses include rodents, shrews, moles, and bats. Imjin virus (MJNV) is a shrew-borne hantavirus identified from the Ussuri white-toothed shrews (*Crocidura lasiura*) in the Republic of Korea (ROK) and China. We have isolated MJNV and determined its prevalence and molecular diversity in Gyeonggi province, ROK. However, the distribution and phylogeography of MJNV in other regions of ROK remain unknown. A total of 96 *C. lasiura* were captured from Gangwon and Gyeonggi provinces, ROK, during 2011–2014. Among them, four (4.2%) shrews were positive for anti-MJNV IgG and MJNV RNA was detected from nine (9.4%), respectively. Based on the prevalence of MJNV RNA, the preponderance of infected shrews was male and adult, consistent with the gender- and weight-specific prevalence of hantaviruses in other species. We monitored the viral load of MJNV RNA in various tissues of shrews, which would reflect the dynamic infectious status and circulation of MJNV in nature. Our phylogeographic and genomic characterization of MJNV suggested natural occurrences of recombination and reassortment in the virus population. Thus, these findings provide significant insights into the epidemiology, phylogeographic diversity, and dynamic circulation and evolution of shrew-borne hantaviruses.

Hantaviruses (genus *Hantavirus*, family *Bunyaviridae*) are zoonotic, negative-sense, single-stranded RNA viruses containing large (L), medium (M), and small (S) segments that encode RNA-dependent RNA polymerase (RdRp), glycoproteins (Gn and Gc), and nucleocapsid (N) protein, respectively^1^. Hantaviruses cause hemorrhagic fever with renal syndrome (HFRS) with fatality rates of 1–15% in Eurasia and hantavirus pulmonary syndrome (HPS) with mortality rates of 30–50% in Americas^2^,^3^. Natural hosts of hantaviruses include rodents (Rodentia), bats (Chiroptera), and insectivores (Soricomorpha)^4^,^5^,^6^. To date, hantaviruses have been identified in shrews and moles (Eulipotyphla). These viruses include Thottapalayam virus (TPMV) from the Asian house shrew (*Suncus murinus*), Imjin virus (MJNV) from the Ussuri white-toothed shrew (*Crocidura lasiura*), Jeju virus (JJUV) from the Asian lesser white-toothed shrew (*C. shantungensis*), Camp Ripley virus (RPLV) from the northern short-tailed shrew (*Blarina brevicauda*), Cao Bang virus (CBNV) from the Chinese mole shrew (*Anourosorex squamipes*), Boginia virus (BOGV) from the Eurasian water shrew (*Neomys fodiens*), Seewis virus (SWSV) from the Eurasian common shrew (*Sorex araneus*), and Artybash virus (ARTV) from the Laxmann’s shrew (*S. caecutiens*)^7^,^8^,^9^,^10^,^11^,^12^,^13^,^14^,^15^.

In our previous study, we first discovered and isolated a shrew-borne hantavirus, MJNV, from *C. lasiura* captured near Imjin River in Gyeonggi province, Republic of Korea (ROK)^8^. MJNV was also found to circulate in Southeastern China^16^. A phylogeographic study of MJNV revealed the molecular diversity of the virus in a limited area, Gyeonggi province^17^. Infection with MJNV elicited a robust expression of pro-inflammatory cytokines in human macrophages and endothelial cells^18^. In a Syrian hamster model, MJNV infection causes a lethal disease in infants and juveniles, suggesting that MJNV may be pathogenic to humans^19^. However, additional genomic sequences of MJNV strains are required to determine the geographic distribution and molecular prevalence in other areas of ROK, as well as the pathogenicity of MJNV in humans.

Genetic exchanges among viruses give rise to genetic diversities that are the basis for molecular evolution^20^,^21^. Recombination and reassortment are major molecular mechanisms for genetic exchange that results in divergent virus progeny. Previous studies have shown that these genetic events in both RNA and DNA viruses impact their molecular diversity, fitness, and pathogenicity^22^,^23^,^24^. Bunyaviruses have been reported to undergo recombination or reassortment *in vitro* and in nature^25^,^26^,^27^. Our recent study identified an S segment recombinant of Hantaan virus (HTNV) in an HFRS patient specimen^28^. In addition, L segment reassortment of HTNV has been shown to occur in nature and contribute to the geographic diversity of HTNV strains in the ROK^29^. However, whether the molecular genetic events of shrew-borne hantaviruses occur in nature have remained unknown.

This study described the distribution and phylogenetic diversity of MJNV in Gangwon province, ROK. The prevalence of MJNV from 96 shrews was comparable between Gangwon and Gyeonggi provinces. There was a clear preponderance of males and adults among MJNV-infected *C. lasiura*, showing the gender and weight-specific prevalence of hantavirus infection reported in other species of shrews and rodents. Genomic sequences of MJNV were obtained from three (75.0%) of four anti-MJNV IgG seropositive shrews and six (6.5%) of 92 seronegative shrews. Additionally, the entire coding sequence of MJNV 10–8 tripartite RNA was obtained since it was identified in 2010. In an analysis of MJNV RNA in the various tissues of seropositive and seronegative shrews, threshold cycles (Ct) values demonstrated a high viral load (low Ct-value) and divergent distribution of MJNV RNA in IFA^+^PCR^+^ (seropositive and PCR positive) shrews by reverse-transcription quantitative PCR (RT-qPCR) using MJNV M segment-specific primers. Of the IFA^−^PCR^+^ (seronegative but PCR positive) shrews, Cl 12–2 and 14–78 harboured a low amount (high Ct-value) of MJNV RNA in different tissues, whereas MJNV RNA was observed only in the lungs of Cl 14–37, 14–42, and 14–70. Phylogenetic analyses of partial genomic sequences of MJNV tripartite RNA demonstrated the well-supported genetic diversity of MJNV in Gangwon and Gyeonggi provinces. Analyses of MJNV open reading frames (ORFs) suggested that recombination and reassortment naturally occurred in the virus population. MJNV 12–2 showed an evidence of L segment recombination, and MJNV strains from Yeoncheon were very likely M segment reassortants. In conclusion, these observations provide better understandings of the epidemiology, phylogeographic diversity, and dynamic circulation and genetic exchanges of shrew-borne hantaviruses in natural reservoirs.

## Methods

### Ethics statement

Trapping of animals was approved by US Forces Korea (USFK) in accordance with USFK Regulation 40–1 “Prevention, Surveillance, and Treatment of Hemorrhagic Fever with Renal Syndrome”. All procedures and handling of animals were conducted under an approved protocol by the Korea University Institutional Animal Care and Use Committee (KUIACUC, #2010-212).

### Trapping and autopsy

**Figure 1:**
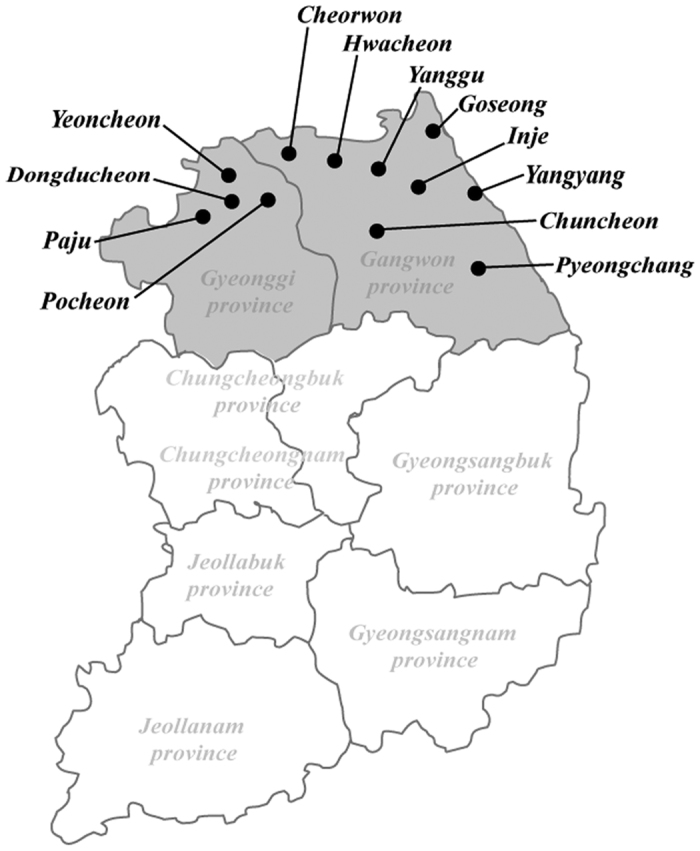
A map of the Republic of Korea showing trapping sites for the Ussuri white-toothed shrews (*Crocidura lasiura*) from 2011 to 2014. A map shows the location of Cheorwon, Hwacheon, Inje, Pyeongchang, Chuncheon, Goseong, Yanggu, and Yangyang in Gangwon province; and Yeoncheon, Paju, and Pocheon in Gyeonggi province. (We used Adobe Illustrator CS6 (http://www.adobe.com/products/illustrator.html) to create the map).

Shrews and rodents, a total of 96 *C. lasiura*, 14 *C. shantungensis*, 921 *Apodemus agrarius*, 4 *A. peninsulae*, 10 *Myodes regulus*, 16 *Micromys minutus*, 2 *Microtus fortis*, 1 *Tamias sibiricus*, and 16 *Tscherskia triton*, were captured in ROK from 2011 to 2014 using Sherman traps (8 by 9 by 23 cm; H. B. Sherman, Tallahassee, FL, USA) baited with peanut butter-covered hardtack. Field trappings were conducted for three days and two nights. For each day, a total of 100 traps were set at intervals of approximately 1 to 2 m. Trapping sites were shown in the Fig. 1; Cheorwon (38°10′19.94′′N 127°18′22.44′′E; 38°10′39.13′′N 127°14′19.42′′E), Chuncheon (37°52′52.72′′N 127°43′48.07′′E), Goseong (38°13′46.39′′N 128°33′14.48′′E), Hwacheon (38°02′55.04′′N 127°32′13.07′′E; 38°06′6.40′′N 127°45′29.93′′E), Inje (38°04′38.55′′N 128°31′24.74′′E; 38°04′34.09′′N 128°15′02.53′′E), Pyeongchang (37°39′57.24′′N 128°35′58.09E; 37°40′10.08′′N 128°35′23.08′′E; 37°40′11.50′′N 128°35′27.90′′E), Yanggu (38°06′51.00′′N 127°58′19.34′′E; 38°16′12.21′′N 128°08′42.88′′E), and Yangyang (38°04′22.63′′N 128°28′58.68′′E; 38°04′55.37′′N 128°26′54.44′′E) in Gangwon province and Dongducheon (37°55′27.37′′N 127°03′57.81′′E), Yeoncheon (38°05′56.02′′N 127°01′0.55′′E; 38°02′53.99′′N 127°06′28.24′′E), Paju (37°58′28.98′′N 126°51′05.12′′E; 37°52′44.46′′N 126°55′39.39′′E; 37°56′36.45′′N 126°54′59.77′′E; 37°58′37.0′′N 126°50′44.1′′E; 37°53′14.8′′N 126°55′55.3′′E), and Pocheon (38°01′41.0′′N 127°12′35.1′′E; 38°03′22.40′′N 127°09′8.59′′E) in Gyeonggi province. The trapping success rate for animals captured during 2011–2014, ROK, was shown in the Table 1. For this study, blood samples were collected from *C. lasiura* via cardiac puncture, and serum was isolated by centrifugation for 5 min at 4 °C. Lungs, livers, kidneys, and spleens were collected and stored at −80 °C.

### Indirect immunofluorescent antibody (IFA) test

Sera were initially diluted 1:32 in phosphate-buffered saline (PBS) and examined for anti-MJNV IgG antibody. The diluted sera were added to wells of acetone-fixed Vero E6 cells infected with MJNV, and the slides were incubated for 30 min at 37 °C. After washing twice, fluorescein isothiocyanate-conjugated goat antibody to rat and mouse IgG antibodies (MP Bio Inc., CA, USA) was added, and the slides were incubated at 37 °C for 30 min. After washing three times, virus-specific fluorescence was evaluated using a fluorescent microscope (Axioscope, Zeiss, Berlin, Germany).

### Reverse transcription-polymerase chain reaction (RT-PCR) and DNA sequencing

Total RNA was extracted from lung tissues of seropositive and seronegative shrews using TRI Reagent Solution (AMBION Inc., Austin, Texas, USA). Using random hexamers, cDNA was synthesized using M-MLV reverse transcriptase (Promega, Madison, WI, USA) or a high capacity RNA-to-cDNA kit (Applied Biosystems, Foster City, CA, USA). First and nested PCRs were performed in a 25-μl reaction mixture containing 2.5U of Ex Taq DNA polymerase (TaKaRa BIO Inc., Shiga, Japan), 2 μg of cDNA, 10 pM of each primer, and 200 μM dNTP (Elpis Biotech, Daejeon, ROK). Oligonucleotide primer sequences for the nested PCR were MJN-L942F (outer): 5′-TCAGAATATAAACCTATGAC-3′, MJN-L1636R (outer): 5′-GATCAACAAACCCATATC-3′, MJN-L942F (inner) and MJN-L1612R (inner): 5′-CTTACATGAGCAGTGGCTA-3′ for the L segment; MJN-M2235F (outer): 5′-CATGGAAGAGTGCAACATGT-3′ and MJN-M2855R (outer): 5′-TATGGTCCCTAGATGTACT-3′, MJV-M2235F (inner) and MJN-M2805R (inner): 5′-TCTATAATAGGATCAGTCAT-3′ for the M segment; MJN-S350F (outer): 5′-GTTGAAGAAGGTGAYTATYTG-3′, MJN-S1102R (outer): 5′-TATRTCCTGCATTAATGCAA-3′, MJN-S350F (inner) and MJN-S1023R (inner): 5′-GGTGCATTYGCAAAAATCCA-3′ for the S segment. Initial denaturation was performed at 95 °C for 5 min, followed by 15 cycles of denaturation at 94 °C for 30 sec, annealing at 50 °C for 40 sec, elongation at 72 °C for 1 min, and then 25 cycles of denaturation at 94 °C for 40 sec, annealing at 52 °C for 40 sec, and elongation at 72 °C for 1 min (ProFlex PCR System, Life Technology, CA, USA). PCR products were purified by LaboPass PCR purification kit (Cosmo Genetech, Seoul, ROK), and sequencing was performed in both directions of each PCR product using a BigDye Terminator v3.1 Cycle Sequencing Kit (Applied Biosystems) on an automated sequencer (ABI 3730XL DNA Analyzer, Applied Biosystems).

### Sequencing analysis of the shrew mitochondrial *cytochrome b* gene

To identify the species of shrews, mitochondrial DNA *cytochrome b* genes of shrews were amplified by PCR and phylogenetically analysed using MEGA 5.2^30^.

### Quantitative real-time PCR

Total RNA was reverse-transcribed using a high-capacity RNA-to-cDNA Kit (Applied Biosystems), with each 10-μL reaction containing 1 μg of total RNA from lungs, livers, kidneys, and spleens. Using a SYBR Green PCR Master Mix (Applied Biosystems) on a StepOne Real-Time PCR System (Applied Biosystems), reactions were performed at a cycle of 95 °C for 10 min, followed by 45 cycles at 95 °C for 15 s, 60 °C for 1 min. Primer sequences targeting MJNV M segment were MJNV-M828F: 5′–AATTTAGGAAAAATCCACAAGGTG–3′ and MJNV-M948R: 5′–TTGAATGCTGCTAGGGTGTTT–3′.

### Phylogenetic analysis

Viral genomic sequences were aligned and edited using the MUSCLE algorithm. Phylogenetic trees were generated by neighbour joining (NJ) and maximum likelihood (ML) methods (MEGA 5.2)^31^. Support for the topologies was assessed by bootstrapping for 1,000 iterations^9^. In addition, MrBayes 3.2.2 program was used for a Bayesian analysis. Markov chain Monte Carlo (MCMC) runs with 6 chains of 20,000,000 generations were sampled every 1,000 generations after a 25% burn-in^32^. Maximum clade credibility trees were prepared in FigTree version 1.4.0.

### Analyses of genomic recombination and reassortment

Alignments of the concatenated MJNV L, M, and S segment ORFs were analysed using RDP, GENECONV, MAXCHI, CHIMAERA, 3SEQ, BOOTSCAN, and SISCAN in the Recombination Detection Program 4 (RDP4) package^33^. Recombination and reassortment events were significantly suggested by RDP4 if at least two criteria were satisfied; the *P*-value (*p*) was under 0.05 and the RDP recombination consensus score (RDPRCS) was over 0.6^27^. Recombination and reassortment events were considered possible when *p* was under 0.05 and the RDPRCS was between 0.4 and 0.6. The likelihood of recombination and reassortment events was considered insignificant when the RDPRCS was under 0.4 with *p* < 0.05. Subsequently, phylogenetic relationships were reconstructed for each genetic event using the ML method in MEGA 5.2.

## Results

A total of 96 Ussuri white-toothed shrews (*C. lasiura*) were captured in Gangwon and Gyeonggi provinces from 2011 to 2014. Using sera from the shrews, serological tests showed that four (4.2%) of 96 samples were positive for anti-MJNV IgG, with three from Gangwon province and one from Gyeonggi province (Table 2). There was no seropositive *C. lasiura* for rodent-borne hantaviruses including HTNV and Seoul virus (SEOV). Partial MJNV L (coordinates 962–1,593 nt) and M (coordinates 2,252–2,784 nt) sequences were detected in nine (9.4%) out of 96 shrews. Among them, three (75.0%) of four seropositive and six (6.5%) of 92 seronegative shrews were positive for the MJNV L and/or M segments, respectively. Seven (17.1%) of 41 males and two (3.6%) of 55 females harboured MJNV RNA. The prevalence of MJNV in the shrews showed heavier animals (≥9.0 g) were infected with MJNV, but there was no positivity of MJNV infection under the animals of 9.0 g. During 2011–2014, most of *C. lasiura* were captured and all of MJNV-positive shrews were observed in autumn. MJNV was not detected from nine of *C. lasiura* collected in spring and summer. Table 3 summarizes the characteristics of MJNV RNA-positive shrews and the nucleotide sequence positions of MJNV RNA obtained in lung tissues of the shrews. The entire coding region of the MJNV L, M, and S segments was sequenced from seven out of nine MJNV-infected shrew samples. In addition, the entire coding nucleotide sequences of MJNV 10–8, identified in 2010^17^, were complete in this study. The genomic sequences of MJNV deposited in GenBank (Accession number: KX779118-KX779145).

**Figure 2:**
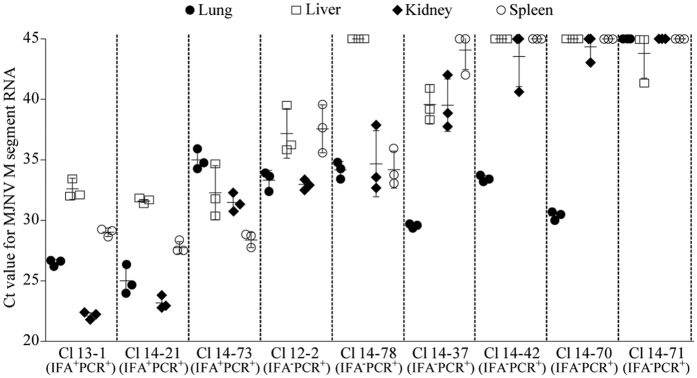
Measurement of threshold cycle (Ct) values of Imjin virus (MJNV) RNA genome in MJNV- infected *Crocidura lasiura* tissues. RT-qPCR was performed to amplify the MJNV M segment in *C. lasiura* tissues, including lungs, livers, kidneys, and spleens. IFA^+^ PCR^+^ indicates anti-MJNV IgG seropositive and MJNV RNA positive shrews (n = 3); IFA^−^ PCR^+^ indicates anti-MJNV IgG seronegative and MJNV RNA positive shrews (n = 6). The vertical axis shows the Ct value for amplification of the MJNV M segment.

To determine the amount of MJNV RNA in IFA^+^PCR^+^ and IFA^−^PCR^+^ *C. lasiura*, real-time PCR was performed on samples from various tissues, including lungs, livers, kidneys, and spleens (Fig. 2). The Ct values of the RT-qPCR to detect MJNV M segment from Cl 13–1, 14–21, and 14–73 (IFA^+^PCR^+^) were overall low (high viral loads) and varied in the different tissues, demonstrating a dynamic MJNV infection in hosts. Cl 12–2 (IFA^−^PCR^+^) showed the presence of MJNV RNA in all tissues, whereas all tissues, except for the liver, from Cl 14–78 (IFA^−^PCR^+^) contained the comparable viral loads of MJNV RNA. The IFA^−^PCR^+^ shrews, Cl 14–37, 14–42 and 14–70, showed the highest MJNV RNA loads in their lungs, whereas there were very low amounts of viral RNA in their livers, kidneys, and spleens. The amount of MJNV RNA in Cl 14–71 (IFA^−^PCR^+^) was likely less than the limit of detection of the assay.

**Figure 3:**
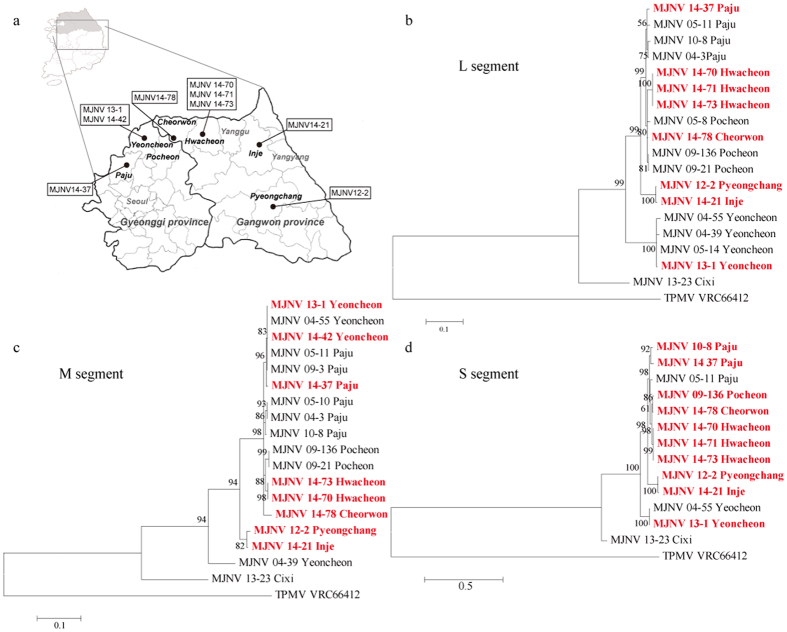
Phylogenetic analyses of the partial sequences of Imjin virus (MJNV) L, M, and S segments in Gangwon and Gyeonggi provinces. (**a**) Geographic locations (red circles) of the Republic of Korea indicate where the genomic sequences of MJNV were acquired. Phylogenetic trees were generated by ML methods, based on the (**b**) 632-nucleotide L segment (coordinates 962–1,593 nt), (**c**) 531-nucleotide M segment (coordinates 2,252–2,784 nt), and (**d**) 608-nucleotide S segment (coordinates 405–1,012 nt) of MJNV strains. Red colour indicates the newly obtained MJNV strains during 2011–2014. The phylogenetic positions of MJNV strains are shown in relationship to representative shrew-borne hantaviruses, including Imjin virus from China (MJNV 13–23 Cixi L segment, KJ420567; M segment, KJ420541; S segment, KJ420559) and Thottapalayam virus (TPMV VRC66412 L segment, EU001330; M segment, DQ825771; S segment, NC_010704). (We used Adobe Illustrator CS6 (http://www.adobe.com/products/illustrator.html) to create the map)

To investigate the genetic diversity of MJNV in Gangwon and Gyeonggi provinces, phylogenetic trees were generated by NJ, ML, and Bayesian methods. The geographic locations of MJNV strains collected in this study are shown in the Fig. 3a. The phylogenetic analysis of the partial MJNV L segment, coordinated to 962–1,593 nt, demonstrated well-supported geographical clusters (Fig. 3b). In Gangwon province, sequences of MJNV strains from Hwacheon clustered with each other. MJNV 12–2 from Pyeongchang formed an independent genetic lineage with MJNV 14–21 from Inje. MJNV 14–78 from Cheorwon grouped with the MJNV strains from Pocheon, Gyeonggi province. In Gyeonggi province, MJNV strains from Paju formed a distinct geographic cluster. MJNV strains from Yeoncheon seemed to be differentiated from all other MJNV strains in ROK. The L segment of MJNV strains differed by 0.0–13.0% and 0.0–4.4% at the nucleotide and amino acid levels, respectively (Table S1). The partial MJNV M sequences (coordinates 2,252–2,784 nt) detected in the shrews from Gangwon and Gyeonggi provinces showed that MJNV strains from Hwacheon and Pocheon formed geographical clusters (Fig. 3c). The partial M sequence of MJNV 12–2 from Pyeongchang formed a distinct genetic lineage with MJNV 14–21 from Inje. MJNV 04–3, 05–10, and 10–8 from Paju, Gyeonggi province, were included in a single genetic lineage, whereas MJNV 05–11, 09–3, and 14–37 grouped phylogenetically with the MJNV strains from Yeoncheon. The nucleotide and amino acid sequences of the MJNV M segment showed differences of 0.0–11.7% and 0.0–2.3%, respectively (Table S2). Phylogenetic analysis of the partial MJNV S segment, coordinated to 405–1,012 nt, indicated that MJNV strains from Hwacheon formed a geographic cluster (Fig. 3d). Consistent with the partial MJNV L and M sequences, partial S sequences of MJNV 12–2 from Pyeongchang grouped phylogenetically with MJNV 14–21 from Inje in Gangwon province. MJNV 14–78 from Cheorwon was closely related to MJNV 09–136 from Pocheon. In Gyeonggi province, MJNV 10–8 and 14–37 from Paju clustered together. The genetic lineage comprising MJNV strains from Yeoncheon was distinct from all other MJNV strains. The differences of nucleotide and amino acid sequences of the partial MJNV S segment differed by 0.0–12.0% and 0.0–1.5%, respectively (Table S3).

**Figure 4:**
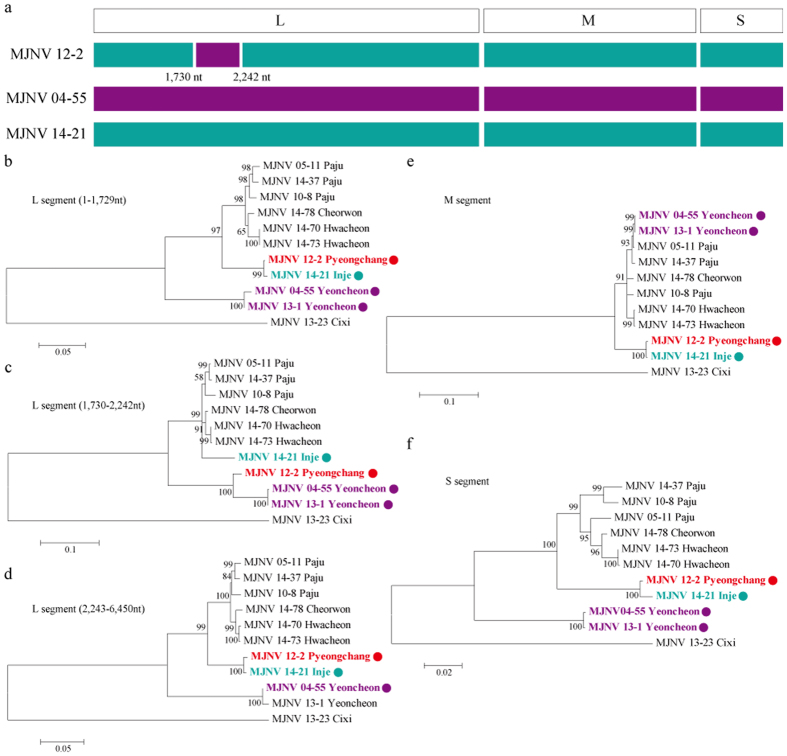
Evidence of a recombination event in Imjin virus (MJNV) strain MJNV 12–2 from Pyeongchang, Republic of Korea. (**a**) The Bootscan plot was based on a pairwise distance model using the RDP4 algorithm. Green and violet colours represent the comparison of MJNV 12–2 (Pyeongchang) to MJNV 14–21 (Inje) and MJNV 04–55 (Yeoncheon), respectively. Bootscan support values above 70.0% (cut-off value) were considered significant. (**b**–**f**) Phylogenetic trees are shown for the L (45–1,774 nt; 1,775–2,287 nt; 2,288–6,494 nt), M (41–3,403 nt), and S (68–1,378 nt) segments of the recombinant MJNV 12–2 using ML method.

To evaluate recombination and reassortment events, sequences of the tripartite ORFs from MJNV genomes were concatenated, aligned, and analysed using RDP 4.0. The partial sequence of MJNV 12–2 L segment, originated from Pyeongchang, was very likely to represent a recombination with MJNV 04–55 from Yeoncheon (Fig. 4a). The *p*-values of the analysis ranged from 2.824E-5 to 7.821E-30, and the RDPRCS of MJNV12-2 was 0.725. Using MJNV strains representing a recombinant, parents, and in- and out-groups, phylogenetic trees of the MJNV L, M, and S segments were generated. The recombined region (coordinated 1,775 to 2,287 nt) of the MJNV 12-2 L segment clustered with MJNV strains from Yeoncheon, whereas the rest of the MJNV 12-2 L segment (coordinated 45 to 1,774 nt and 2,288 to 6,494 nt) clustered with MJNV 14–21 from Inje (Fig. 4b–d). The ORF sequences of MJNV 12–2 M and S segments clustered with those of MJNV 14–21 (Fig. 4e and f). These results indicate that MJNV12-2 may be a recombinant because of a partial exchange of the L segment in nature.

**Figure 5:**
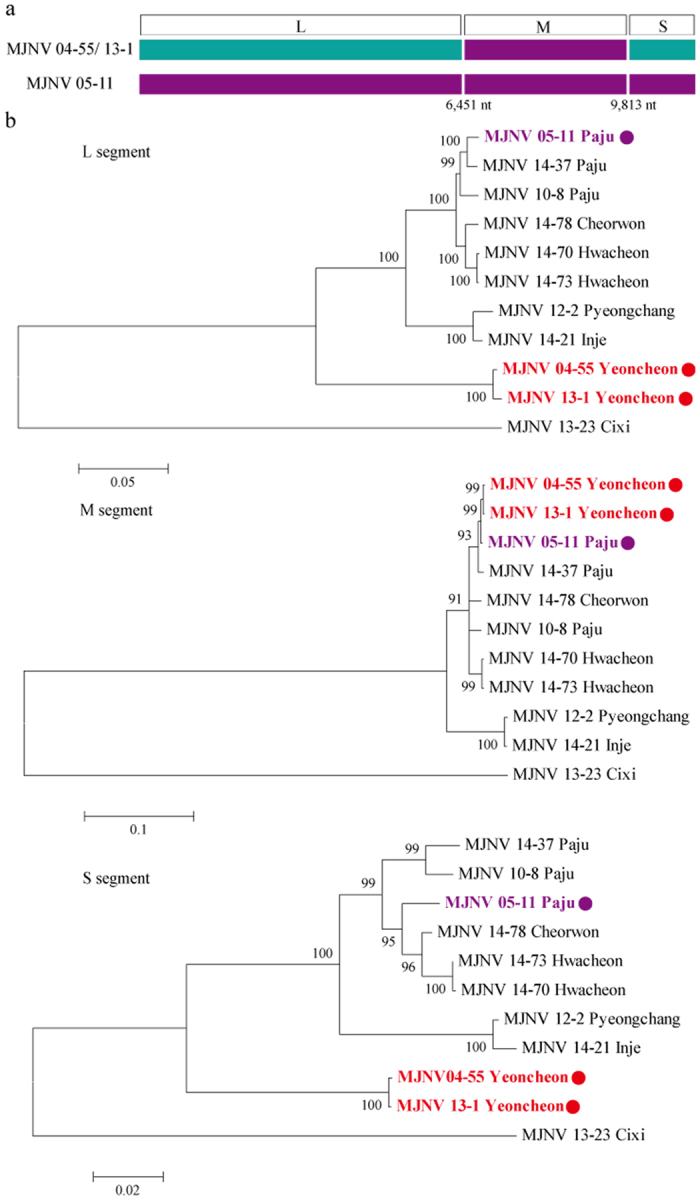
Evidence of a natural reassortment of Imjin virus (MJNV) strains in Yeoncheon, Republic of Korea. (**a**) The Bootscan plot was generated on a pairwise distance model using the RDP4 algorithm. Green colour indicates the genomic composition of MJNV 04–55 and 13–1 from Yeoncheon, and violet colour indicates the genomic composition of MJNV 05–11 from Paju. Bootscan support values above 70.0% (cut-off value) were considered significant. (**b**) Phylogenetic trees were generated for the L, M, and S segments of the reassortant MJNV 04–55 and 13–1.

The RDP4 analysis suggested that the genomic configuration of MJNV 04–55 and 13–1, identified from Yeoncheon, was likely to represent the reassortment of M segments with MJNV 05–11 and 14–37 from Paju (Fig. 5a). The *p*-value from the analysis was under 0.05, and the RDPRCS of MJNV strains from Yeoncheon was 0.548. The results satisfied the criteria to suggest a reassortment event in the MJNV M segment. The phylogenetic trees, including reassortants, parents, and in- and out-groups, demonstrate that the M segments of MJNV strains from Yeoncheon clustered with those from Paju, whereas the L and S segments formed a distinct genetic lineage (Fig. 5b).

## Discussion

We have demonstrated the isolation, characterization, and molecular diversity of MJNV strains collected from *C. lasiura* in Gyeonggi province, ROK^9^,^17^. The previous study described serological and molecular prevalence of MJNV from the shrews in the regions, ROK. Between 2004–2010 and 2011–2014 studies, the seroprevalence of MJNV infection was maintained during a year, demonstrating about 9.0%, 13.5%, 8.0%, and 2.5% for spring, summer, autumn, and winter, respectively. This observation suggests that the circulation of MJNV is enzootic rather than epizootic among shrews.

HTNV, a rodent-borne hantavirus, causes HFRS in the highly endemic areas of ROK and China^34^,^35^. The prevalence of HTNV in Gyeonggi province was found to be higher than that in Gangwon province, corresponding to higher HFRS incidences^36^. We reported that seronegative rodents in the highly HFRS-endemic area of Gyeonggi, but not Gangwon province, harboured HTNV-specific genes^37^. However, the prevalence of MJNV in Gangwon and Gyeonggi provinces was statistically insignificant (Fisher’s exact test). We observed MJNV-specific RNA in the seronegative shrews, suggesting active circulation of MJNV in the regions. Recently, Patrick Heinemann *et al*. reported that African shrew-borne hantaviruses might cause human infections based on serological data^38^. The infection and pathogenicity of MJNV to humans remain to be investigated.

Rodent-borne hantaviruses, e.g. HTNV, Sin Nombre virus (SNV), El Moro Canyon virus (EMCV), Puumala virus (PUUV), Bayou virus (BAYV), have been reported for the higher prevalence of infection in the male and adult (heavier) natural hosts^39^,^40^,^41^,^42^,^43^. We found that male and adult shrews showed significantly higher prevalence of MJNV infection than female (Fisher’s exact test, *p* < 0.05) and youth shrews. The preferential male infection with MJNV suggests that the horizontal transmission of MJNV may occur via aggressive behaviors such as biting or fighting. The dominant prevalence of adult infection with MJNV indicates that reproductive activities may be associated with the transmission of MJNV. The breeding of *C. lasiura* occurs during summer and autumn^44^. The detection of MJNV RNA in shrews was observed in September and November, suggesting the breeding may be one of transmission modes for MJNV.

To examine the viral load of MJNV in shrews, the amount of MJNV was determined from various tissues of IFA^+^PCR^+^ and IFA^−^PCR^+^ animals. IFA^+^PCR^+^ samples showed higher viral loads of MJNV RNA in the tissues, including lungs, kidneys, livers, and spleens. For the three IFA^−^PCR^+^ samples, MJNV RNA was the highest in the lung, reflecting an early phase of infection with MJNV in the endemic areas during autumn (September and November)^45^. An early phase of infection may be explained by MJNV replication primarily in the lung before MJNV-specific IgG antibodies were generated^46^,^47^. The viral RNA loads in the different tissues may reflect the dynamic infectious status and circulation of MJNV among the shrews in autumn.

Genetic exchanges, e.g., recombination and reassortment, promote genetic diversities for viral evolution in nature^24^. These exchanges have influenced fitness, transmission, and pathogenicity of the virus^48^. A recent study demonstrated that the reassortment of PB1, PB2, and HA of influenza B virus contributed to its fitness and replication^49^. Recombination and reassortment of bunyaviruses have been observed in nature and *in vitro*^50^,^51^,^52^. A previous study described M segment recombinants in the HTNV population of southwestern China^53^. Other hantaviruses, such as Tula virus, PUUV, Andes virus, and SEOV, exhibited evidences of the S segment recombination in natural reservoirs or *in vitro*^23^,^26^,^54^,^55^,^56^. SNV, a family of *Bunyaviridae*, has shown the evidence of an M segment reassortment in nature and *in vitro*^52^. Recently, HTNV L segment reassortment was found to occur naturally in Gyeonggi and Gangwon provinces, ROK^29^. In this study, both recombination and reassortment were detected in the MJNV population. Recombinant MJNV 12–2, identified from Pyeongchang, contained a heterogeneous partial L segment sequence (coordinated to 1,775–2,287) that clustered with MJNV 04–55 from Yeoncheon. However, the rest of the L segment and whole M and S segments of MJNV 12–2 formed a genetic lineage with MJNV 14–21 from Inje. This configuration of the MJNV tripartite genomes may exhibit the recombination of L segment in nature. In addition, the M segment of MJNV 04–55 and 13–1 from Yeoncheon grouped with that of MJNV 05–11 from Paju, whereas their L and S segments phylogenetically formed a segregated lineage. This genomic configuration suggested that MJNV strains in Yeoncheon contained a genome organization compatible with reassortment of the M segment. We observed the L segment reassortant and S segment recombinant of HTNV infected humans and caused HFRS, respectively^28^. Dobrava-Belgrade virus (DOBV) showed the reassortment of M segment between avirulent strain DOBV-Aa and virulent strain DOBV-Af *in vitro*^57^. Thus, further studies are required to understand the biological and ecological consequences of recombination or reassortment in the MJNV population.

In conclusion, this study first characterized the geographic prevalence, phylogenetic diversity, and dynamic circulation and molecular evolution of MJNV from *C. lasiura* collected in Gangwon and Gyeonggi provinces, ROK. The preferential infection of MJNV was described in male and adult shrews. Based on 28 partial MJNV sequences, including 24 ORF genomic sequences, the phylogenetic analyses indicated geographic diversity of MJNV in the regions. Profiling of MJNV RNA in various tissues of the shrew specimens may reflect the dynamic circulation of MJNV in natural reservoirs. Evidences for the occurrence of recombination and reassortment suggest active genetic exchanges of shrew-borne hantaviruses in nature. These findings provide broad and deep insights into the epidemiology, virus-host interaction, and molecular evolution of hantaviruses in soricid hosts.

## Additional Information

**How to cite this article:** Lee, S.-H. *et al*. Dynamic Circulation and Genetic Exchange of a Shrew-borne Hantavirus, Imjin virus, in the Republic of Korea. *Sci. Rep.* **7**, 44369; doi: 10.1038/srep44369 (2017).

**Publisher's note:** Springer Nature remains neutral with regard to jurisdictional claims in published maps and institutional affiliations.

## Supplementary Material

Supplementary Information

## Figures and Tables

**Table 1:** The total number and trapping success rate of animals captured during 2011–2014, Republic of Korea.

Species	Number of collection	Trapping success rate (%)*
*Apodemus agrarius*	921	18.4
*Apodemus peninsulae*	4	0.1
*Crocidura lasiura*	96	1.9
*Crocidura shantungensis*	14	0.3
*Myodes regulus*	10	0.2
*Micromys minutus*	16	0.3
*Microtus fortis*	2	0.04
*Tamias sibiricus*	1	0.02
*Tscherskia triton*	16	0.3
Total	1,080	21.6

*Trapping success rate = the total number of collection/the total number of traps** × 100. **The total number of traps = 200 (the number of traps per field trip) × 25 (the total number of field trips).

**Table 2:** Serological and molecular prevalence of Imjin virus (MJNV) for region, sex, weight, and season in the ROK, 2011–2014.

Categories	Seropositve Rate for anti-MJNV IgG antibody (%)	MJNV RNA positivity (%)*	
		Seropositive	Seronegative
Region (n = 96)			
Gangwon province	3/67 (4.5%)	2/3 (66.7%)	4/64 (6.3%)
Gyeonggi province	1/29 (3.5%)	1/1 (100.0%)	2/28 (7.1%)
Sex (n = 96)			
Males	4/41 (9.8%)	3/4 (75%)	4/37 (10.8%)
Females	0/55 (0.0%)	0 (0.0%)	2/55 (3.6%)
Weight (n = 96)			
≤5.5 g	0/4 (0.0%)	0 (0.0%)	0/4 (0.0%)
5.6–9.9 g	0/32 (0.0%)	0 (0.0%)	1/32 (3.1%)
10–14.9 g	3/46 (6.5%)	2/3 (66.7%)	5/43 (11.6%)
15–19.5 g	1/14 (7.1%)	1/1 (100.0%)	0/13 (0.0%)
Season (n = 96)			
Spring (Mar-May)	0/4 (0.0%)	0 (0.0%)	0/4 (0.0%)
Summer (Jun-Aug)	0/5 (0.0%)	0 (0.0%)	0/5 (0.0%)
Autumn (Sep-Nov)	4/87 (4.6%)	3/4 (75.0%)	6/83 (7.2%)
Winter (Dec-Feb)**			

*The positive rate of MJNV RNA indicates the detection of partial L segment (coordinates 962–1,593 nt) and/or M segment (coordinates 2,252–2,784 nt) by RT-PCR and Sanger-sequencing. Total positivity of the RT-PCR was nine (9.4%) out of 96 shrew samples. **There was no trapping in winter, 2011–2014.

**Table 3:** Characteristics of Imjin virus (MJNV)-infected *Crocidura lasiura* and the nucleotide sequence of MJNV tripartite RNA acquired in the study.

Sample	Site (city/province)	Trapping date	Sex	Weight (g)	Vital	anti-MJNV IgG antibody test (IFA)	Nucleotide (nt) position of MJNV RNA		
							L segment	M segment	S segment
10–8^a^,^b^	Paju/Gyeonggi	May 12, 2010	Male	15.0	Alive	+	33–6,528	22–3,446	31–1,545
12–2^a^	Pyeongchang/Gangwon	Sep. 13, 2012	Female	12.0	Dead	−	41–6,528	22–3,446	31–1,545
13–1^a^	Yeoncheon/Gyeonggi	Sep. 12, 2013	Male	19.5	Dead	+	41–6,528	33–3,419	51–1,545
14–21^a^	Inje/Gangwon	Sep. 19, 2014	Male	10.5	Dead	+	24–6,508	22–3,446	31–1,545
14–37^a^	Paju/Gyeonggi	Sep. 24, 2014	Female	10.0	Alive	−	34–6,527	34–3,446	31–1,545
14–42	Yeoncheon/Gyeonggi	Sep. 25, 2014	Male	13.0	Alive	−	·	2,252–2,784	1,140–1,545
14–70^a^	Hwacheon/Gangwon	Nov. 5, 2014	Male	9.0	Dead	−	34–6,527	34–3,419	51–1,545
14–71	Hwacheon/Gangwon	Nov. 5, 2014	Male	10.5	Dead	−	962–2,267	·	371–1,012
14–73^a^	Hwacheon/Gangwon	Nov. 5, 2014	Male	10.5	Dead	+	41–6,528	33–3,446	41–1,545
14–78^a^	Cheorwon/Gangwon	Nov. 13, 2014	Male	13.0	Dead	−	41–6,508	22–3,446	41–1,545

^a^Entire ORF sequences were completely obtained. GenBank (Accession number: KX779118-KX779145).

^b^Partial genomic sequences of MJNV 10–8 was identified in 2010^17^. Entire coding sequences of the MJNV 10–8 were complete for this study.
