# Supplementary material for: Dynamic Circulation and Genetic Exchange of a Shrew-borne Hantavirus, Imjin virus, in the Republic of Korea
Source: Sci Rep. 2017 Mar 15;7:44369. doi: 10.1038/srep44369 (PMC5353647; doi:10.1038/srep44369)
Supplement: Supplementary Information [file srep44369-s1.doc]

**Supplementary information**

**Dynamic Circulation and Genetic Exchange of a Shrew-borne Hantavirus, Imjin virus, in the Republic of Korea**

**Author information**

Seung-Ho Lee^1,†^, Won-Keun Kim^1,†^, Jin Sun No^1^, Jeong-Ah Kim^1^, Jin Il Kim^1^, Se Hun Gu^2^, Heung-Chul Kim^3^, Terry A. Klein^4^, Manseong Park^1^ and Jin-Won Song^1^,*

^1^Department of Microbiology, College of Medicine, Korea University, Seoul, Republic of Korea 02841

^2^5th R&D Institute, Agency for Defense Development, Daejeon, Republic of Korea 34186

^3^5th Medical Detachment, 168th Multifunctional Medical Battalion, 65th Medical Brigade, Unit 15247, APO AP 96205-5247, United States of America

^4^Public Health Command District-Korea (Provisional), 65th Medical Brigade, Unit 15281, APO AP 96205-5281, United States of America

^†^ These authors contributed equally to this study.

*Corresponding author

**Table S1. Percent nucleotide and amino acid sequence similarities of partial MJNV L segments detected in *Crocidura lasiura* from Gangwon and Gyeonggi provinces (Numbers above the diagonal represent nucleotide distances (expressed as a percentage) and numbers below the diagonal represent amino acid distances).**

| **Strain** | **MJNV**  **04-39** | **MJNV**  **04-55** | **MJNV**  **05-14** | **MJNV**  **13-1** | **MJNV**  **04-3** | **MJNV**  **05-11** | **MJNV**  **10-8** | **MJNV**  **14-37** | **MJNV**  **05-8** | **MJNV**  **09-21** | **MJNV**  **09-136** | **MJNV**  **12-2** | **MJNV**  **14-21** | **MJNV**  **14-70** | **MJNV**  **14-71** | **MJNV**  **14-73** | **MJNV**  **14-78** |
| --- | --- | --- | --- | --- | --- | --- | --- | --- | --- | --- | --- | --- | --- | --- | --- | --- | --- |
| **MJNV**  **04-39** |  | 98.4 | 99.4 | 99.2 | 89.2 | 88.6 | 88.9 | 88.8 | 89.2 | 89.2 | 89.1 | 87.8 | 87.3 | 88.1 | 88.1 | 88.1 | 88.9 |
| **MJNV**  **04-55** | 97.6 |  | 99.0 | 98.9 | 88.9 | 88.3 | 88.6 | 88.4 | 88.9 | 88.9 | 88.8 | 87.5 | 87.0 | 87.8 | 87.8 | 87.8 | 88.6 |
| **MJNV**  **05-14** | 99.5 | 98.1 |  | 99.8 | 89.6 | 88.9 | 89.2 | 89.1 | 89.6 | 89.6 | 89.4 | 88.1 | 87.6 | 88.4 | 88.4 | 88.4 | 89.2 |
| **MJNV**  **13-1** | 99.5 | 98.1 | 100 |  | 89.4 | 88.8 | 89.1 | 88.9 | 89.4 | 89.4 | 89.2 | 88.3 | 87.8 | 88.3 | 88.3 | 88.3 | 89.1 |
| **MJNV**  **04-3** | 98.5 | 97.1 | 99.0 | 99.0 |  | 98.7 | 99.2 | 98.9 | 98.1 | 98.7 | 98.6 | 94.9 | 94.7 | 97.8 | 97.8 | 97.8 | 98.7 |
| **MJNV**  **05-11** | 97.6 | 96.1 | 98.1 | 98.1 | 99.0 |  | 98.4 | 98.6 | 97.8 | 98.4 | 98.2 | 94.1 | 93.9 | 97.3 | 97.3 | 97.3 | 98.4 |
| **MJNV**  **10-8** | 98.1 | 96.6 | 98.5 | 98.5 | 99.5 | 98.5 |  | 98.6 | 97.8 | 98.4 | 98.2 | 94.2 | 94.4 | 97.3 | 97.3 | 97.3 | 98.4 |
| **MJNV**  **14-37** | 98.5 | 97.1 | 99.0 | 99.0 | 100 | 99.0 | 99.5 |  | 97.9 | 98.6 | 98.4 | 94.4 | 94.2 | 97.3 | 97.3 | 97.3 | 98.6 |
| **MJNV**  **05-8** | 98.5 | 97.1 | 99.0 | 99.0 | 100 | 99.0 | 99.5 | 100 |  | 98.7 | 98.9 | 93.9 | 93.7 | 97.8 | 97.8 | 97.8 | 99.0 |
| **MJNV**  **09-21** | 98.5 | 97.1 | 99.0 | 99.0 | 100 | 99.0 | 99.5 | 100 | 100 |  | 99.8 | 94.2 | 94.1 | 98.1 | 98.1 | 98.1 | 99.4 |
| **MJNV**  **09-136** | 98.5 | 97.1 | 99.0 | 99.0 | 100 | 99.0 | 99.5 | 100 | 100 | 100 |  | 94.4 | 94.2 | 98.2 | 98.2 | 98.2 | 99.5 |
| **MJNV**  **12-2** | 99.0 | 97.6 | 99.5 | 99.5 | 99.5 | 98.5 | 99.0 | 99.5 | 99.5 | 99.5 | 99.5 |  | 99.5 | 93.6 | 93.6 | 93.6 | 94.5 |
| **MJNV**  **14-21** | 98.5 | 97.1 | 99.0 | 99.0 | 99.0 | 98.1 | 98.5 | 99.0 | 99.0 | 99.0 | 99.0 | 99.5 |  | 93.4 | 93.4 | 93.4 | 94.4 |
| **MJNV**  **14-70** | 97.1 | 95.6 | 97.6 | 97.6 | 98.5 | 97.6 | 98.1 | 98.5 | 98.5 | 98.5 | 98.5 | 98.1 | 97.6 |  | 100 | 100 | 98.4 |
| **MJNV**  **14-71** | 97.1 | 95.6 | 97.6 | 97.6 | 98.5 | 97.6 | 98.1 | 98.5 | 98.5 | 98.5 | 98.5 | 98.1 | 97.6 | 100 |  | 100 | 98.4 |
| **MJNV**  **14-73** | 97.1 | 95.6 | 97.6 | 97.6 | 98.5 | 97.6 | 98.1 | 98.5 | 98.5 | 98.5 | 98.5 | 98.1 | 97.6 | 100 | 100 |  | 98.4 |
| **MJNV**  **14-78** | 98.5 | 97.1 | 99.0 | 99.0 | 100 | 99.0 | 99.5 | 100 | 100 | 100 | 100 | 99.5 | 99.0 | 98.5 | 98.5 | 98.5 |  |

**Table S2. Percent nucleotide and amino acid sequence similarities of partial MJNV M segments detected in *Crocidura lasiura* from Gangwon and Gyeonggi provinces (Numbers above the diagonal represent nucleotide distances (expressed as a percentage) and numbers below the diagonal represent amino acid distances)**.

| **Strain** | **MJNV**  **04-39** | **MJNV**  **04-55** | **MJNV**  **13-1** | **MJNV**  **14-42** | **MJNV**  **04-3** | **MJNV**  **05-10** | **MJNV**  **05-11** | **MJNV**  **09-3** | **MJNV**  **10-8** | **MJNV**  **14-37** | **MJNV**  **09-21** | **MJNV**  **09-136** | **MJNV**  **12-2** | **MJNV**  **14-21** | **MJNV**  **14-70** | **MJNV**  **14-73** | **MJNV**  **14-78** |
| --- | --- | --- | --- | --- | --- | --- | --- | --- | --- | --- | --- | --- | --- | --- | --- | --- | --- |
| **MJNV**  **04-39** |  | 88.6 | 88.6 | 88.5 | 88.8 | 88.8 | 88.6 | 88.6 | 89.0 | 88.6 | 88.3 | 88.3 | 90.3 | 89.9 | 88.6 | 88.6 | 88.8 |
| **MJNV**  **04-55** | 98.3 |  | 100 | 99.8 | 98.6 | 98.6 | 100 | 100 | 98.7 | 99.5 | 98.2 | 98.2 | 93.5 | 94.1 | 98.4 | 98.4 | 97.7 |
| **MJNV**  **13-1** | 98.3 | 100 |  | 99.8 | 98.6 | 98.6 | 100 | 100 | 98.7 | 99.5 | 98.2 | 98.2 | 93.5 | 94.1 | 98.4 | 98.4 | 97.7 |
| **MJNV**  **14-42** | 98.3 | 100 | 100 |  | 98.4 | 98.4 | 99.8 | 99.8 | 98.6 | 99.3 | 98.0 | 98.0 | 93.3 | 93.9 | 98.2 | 98.2 | 97.5 |
| **MJNV**  **04-3** | 98.3 | 100 | 100 | 100 |  | 100 | 98.6 | 98.6 | 99.5 | 98.4 | 98.2 | 98.2 | 93.3 | 93.9 | 98.4 | 98.4 | 97.7 |
| **MJNV**  **05-10** | 98.3 | 100 | 100 | 100 | 100 |  | 98.6 | 98.6 | 99.5 | 98.4 | 98.2 | 98.2 | 93.3 | 93.9 | 98.4 | 98.4 | 97.7 |
| **MJNV**  **05-11** | 98.3 | 100 | 100 | 100 | 100 | 100 |  | 100 | 98.7 | 99.5 | 98.2 | 98.2 | 93.5 | 94.1 | 98.4 | 98.4 | 97.7 |
| **MJNV**  **09-3** | 98.3 | 100 | 100 | 100 | 100 | 100 | 100 |  | 98.7 | 99.5 | 98.2 | 98.2 | 93.5 | 94.1 | 98.4 | 98.4 | 97.7 |
| **MJNV**  **10-8** | 98.3 | 100 | 100 | 100 | 100 | 100 | 100 | 100 |  | 98.6 | 98.4 | 98.4 | 93.5 | 94.1 | 98.6 | 98.6 | 97.8 |
| **MJNV**  **14-37** | 98.3 | 100 | 100 | 100 | 100 | 100 | 100 | 100 | 100 |  | 98.0 | 98.0 | 93.2 | 93.7 | 98.2 | 98.2 | 97.5 |
| **MJNV**  **09-21** | 97.7 | 99.4 | 99.4 | 99.4 | 99.4 | 99.4 | 99.4 | 99.4 | 99.4 | 99.4 |  | 100 | 92.8 | 93.3 | 98.7 | 98.7 | 97.3 |
| **MJNV**  **09-136** | 97.7 | 99.4 | 99.4 | 99.4 | 99.4 | 99.4 | 99.4 | 99.4 | 99.4 | 99.4 | 100 |  | 92.8 | 93.3 | 98.7 | 98.7 | 97.3 |
| **MJNV**  **12-2** | 98.3 | 100 | 100 | 100 | 100 | 100 | 100 | 100 | 100 | 100 | 99.4 | 99.4 |  | 99.1 | 93.2 | 93.2 | 92.8 |
| **MJNV**  **14-21** | 98.3 | 100 | 100 | 100 | 100 | 100 | 100 | 100 | 100 | 100 | 99.4 | 99.4 | 100 |  | 93.7 | 93.7 | 93.0 |
| **MJNV**  **14-70** | 97.7 | 99.4 | 99.4 | 99.4 | 99.4 | 99.4 | 99.4 | 99.4 | 99.4 | 99.4 | 98.9 | 98.9 | 99.4 | 99.4 |  | 100 | 97.5 |
| **MJNV**  **14-73** | 97.7 | 99.4 | 99.4 | 99.4 | 99.4 | 99.4 | 99.4 | 99.4 | 99.4 | 99.4 | 98.9 | 98.9 | 99.4 | 99.4 | 100 |  | 97.5 |
| **MJNV**  **14-78** | 97.7 | 99.4 | 99.4 | 99.4 | 99.4 | 99.4 | 99.4 | 99.4 | 99.4 | 99.4 | 98.9 | 98.9 | 99.4 | 99.4 | 98.9 | 98.9 |  |

**Table S3. Percent nucleotide and amino acid sequence similarities of partial MJNV S segments detected in *Crocidura lasiura* from Gangwon and Gyeonggi provinces (Numbers above the diagonal represent nucleotide distances (expressed as a percentage) and numbers below the diagonal represent amino acid distances).**

| **Strain** | **MJNV**  **04-55** | **MJNV**  **13-1** | **MJNV**  **05-11** | **MJNV**  **10-8** | **MJNV**  **14-37** | **MJNV**  **09-136** | **MJNV**  **12-2** | **MJNV**  **14-21** | **MJNV**  **14-70** | **MJNV**  **14-71** | **MJNV**  **14-73** | **MJNV**  **14-78** |
| --- | --- | --- | --- | --- | --- | --- | --- | --- | --- | --- | --- | --- |
| **MJNV**  **04-55** |  | 99.8 | 89.7 | 89.5 | 90.3 | 89.8 | 88.3 | 88.0 | 90.0 | 90.0 | 90.0 | 90.1 |
| **MJNV**  **13-1** | 100 |  | 89.8 | 89.7 | 90.5 | 90.0 | 88.5 | 88.2 | 90.1 | 90.1 | 90.1 | 90.3 |
| **MJNV**  **05-11** | 100 | 100 |  | 96.4 | 96.4 | 97.0 | 92.6 | 92.0 | 96.9 | 96.9 | 96.9 | 97.0 |
| **MJNV**  **10-8** | 99.5 | 99.5 | 99.5 |  | 97.2 | 99.5 | 91.8 | 91.1 | 96.1 | 96.1 | 96.1 | 95.9 |
| **MJNV**  **14-37** | 99.5 | 99.5 | 99.5 | 100 |  | 95.2 | 92.0 | 91.6 | 95.4 | 95.4 | 95.4 | 95.2 |
| **MJNV**  **09-136** | 100 | 100 | 100 | 99.5 | 99.5 |  | 92.4 | 91.8 | 98.2 | 98.2 | 98.2 | 99.0 |
| **MJNV**  **12-2** | 99.0 | 99.0 | 99.0 | 98.5 | 98.5 | 99.0 |  | 99. 3 | 92.3 | 92.3 | 92.3 | 92.8 |
| **MJNV**  **14-21** | 99.0 | 99.0 | 99.0 | 98.5 | 98.5 | 99.0 | 100 |  | 91.6 | 91.6 | 91.6 | 92.1 |
| **MJNV**  **14-70** | 100 | 100 | 100 | 99.5 | 99.5 | 100 | 99.0 | 99.0 |  | 100 | 100 | 98.5 |
| **MJNV**  **14-71** | 100 | 100 | 100 | 99.5 | 99.5 | 100 | 99.0 | 99.0 | 100 |  | 100 | 98.5 |
| **MJNV**  **14-73** | 100 | 100 | 100 | 99.5 | 99.5 | 100 | 99.0 | 99.0 | 100 | 100 |  | 98.5 |
| **MJNV**  **14-78** | 99.5 | 99.5 | 99.5 | 99.0 | 99.0 | 99.5 | 98.5 | 98.5 | 99.5 | 99.5 | 99.5 |  |
